# Application of Hybrid Genetic Algorithm Routine in Optimizing Food and Bioengineering Processes

**DOI:** 10.3390/foods5040076

**Published:** 2016-11-09

**Authors:** Jaya Shankar Tumuluru, Richard McCulloch

**Affiliations:** Biofuels Department, Energy and Environment Directorate, Idaho National Laboratory, 750 University Boulevard, MS 3570, Idaho Falls, ID 83415, USA

**Keywords:** hybrid genetic algorithm, optimization, Ackley function, response surface functions, anthocyanin yield, fatty acid methyl ester, xylanase activity

## Abstract

Optimization is a crucial step in the analysis of experimental results. Deterministic methods only converge on local optimums and require exponentially more time as dimensionality increases. Stochastic algorithms are capable of efficiently searching the domain space; however convergence is not guaranteed. This article demonstrates the novelty of the hybrid genetic algorithm (HGA), which combines both stochastic and deterministic routines for improved optimization results. The new hybrid genetic algorithm developed is applied to the Ackley benchmark function as well as case studies in food, biofuel, and biotechnology processes. For each case study, the hybrid genetic algorithm found a better optimum candidate than reported by the sources. In the case of food processing, the hybrid genetic algorithm improved the anthocyanin yield by 6.44%. Optimization of bio-oil production using HGA resulted in a 5.06% higher yield. In the enzyme production process, HGA predicted a 0.39% higher xylanase yield. Hybridization of the genetic algorithm with a deterministic algorithm resulted in an improved optimum compared to statistical methods.

## 1. Introduction

In chemical processes, the process parameters such as temperature, residence time, and pressure, play a major role in determining the process efficiencies and product properties. Tumuluru et al., in their studies [1], indicated that extrusion is an important food manufacturing process. The food properties are dependent on process variables and material properties of the food [2,3,4,5]. In biomass densification using a pellet mill, Tumuluru [6,7,8] indicated that feedstock parameters (moisture content) and process parameters (preheating temperature and die rotational speed) influence the product properties and the energy consumption of the pelleting process.

To understand the effect of process variables on the product properties and further optimization, response surface methodology (RSM) is commonly used. RSM is a combination of mathematical and statistical techniques applied to data obtained from experiments performed according to specific special experimental designs [9] and is probably the most common optimization method. RSM models usually represent data as a polynomial with coefficients determined through least-squares regression. Many researchers have used RSM to understand the effect of process variables and to optimize the product properties [4,10,11,12,13,14,15]. In particular, Shankar et al. [5] state that RSM is a good approach to summarize the trends of process variables for either maximization or minimization of the product quality. These same authors also indicate that the interpretation of RSM results is very complex, especially when optimizing a function with more than three independent variables. In these situations, an RSM solution is often on a saddle point, and thus is not optimized. Stochastic algorithms, such as the genetic algorithm (GA), have the capability of optimizing these complex solutions, where deterministic methods such as RSM fail.

### 1.1. Genetic Algorithm

Genetic algorithms (GAs) have gained importance in optimization due to their ability to find optimized conditions for multi-dimensional complex problems while utilizing minimal resources. This is achieved by the design of GAs, namely simulating natural evolution. In a standard GA a population of possible solutions is used as a pool from which to draw parents. Parents are randomly selected, however, optimum candidates are given preference during the mating routine. Selected parents are crossed to simulate mating, which results in a single new offspring. Random offspring mutation is included to increase the exploration capabilities of the algorithm. Elitism is often included in the GA, which ensures that the best-so-far candidate is preserved and unchanged, in the new offspring generation. This allows the GA to be equal or with a greater optimum than previous generations. By harnessing the mechanisms of evolution, researchers are able to “breed” programs that solve problems even when their structure is not fully understood. GAs make it possible to explore a far greater number of potential solutions to a problem than conventional programs [16]. The main advantages of GAs are (1) GA-based approaches are capable of finding a number of optimal solutions rather than a single solution [17]; (2) GA-based approaches are capable of exploring the search space more thoroughly with a smaller number of performance evaluations than those based on a local search, such as simulated annealing and Tabu search [18]; (3) GA-based approaches are less dependent on selecting good starting points, and they do not require neighborhood definitions [18]; (4) they can solve every optimization problem that can be described with chromosome encoding; (5) they solve problems with multiple solutions; and (6) since the genetic algorithm execution technique is not dependent on the error surface, multi-dimensional, non-differential, non-continuous, and even non-parametrical problems can be solved.

GAs are stochastic in nature, and thus have multiple benefits therein, such as drawing from a population, not a single possible solution, and relying on objective function information instead of topological information such as derivatives, etc. This frees the GA to assess a possible candidate solution at any point in the variable domain space. GAs find extensive applications where the process systems are highly complex and nonlinear [16,19]. Chun [19] discussed the usefulness of heuristic algorithms as the search method for diverse optimization problems. Their studies include a comparison of immune algorithms, genetic algorithms, and evolutionary algorithms on diverse optimization problems and indicated that results of the genetic algorithm are superior to others. The major limitation of the genetic algorithm is that, being a heuristic method, it cannot reach the global optimum and the optimization times are longer. The genetic algorithm cannot be applied to certain problems, called variant problems, which results in poor fitness and the generation of bad chromosomes.

### 1.2. Deterministic Optimization

**Figure 1:**
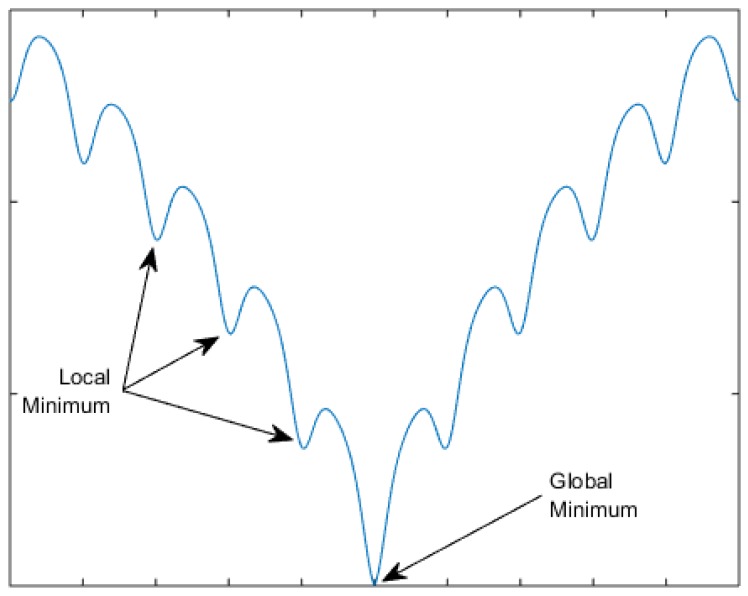
An example of local and global optimum points in function minimization.

On the opposite end of the spectrum, deterministic algorithms are capable of finding the exact optimum for a given problem. The main drawback for deterministic algorithms is that the function must be well defined and well behaved, and even then the algorithm converges locally, not globally. To ensure global convergence, a “brute force” full factorial approach is necessary; however, this quickly becomes unfeasible as dimensionality and variable space increase. Figure 1 indicates the possibility of how local and global search algorithms can get stuck at complex global search spaces. Hybridization of the genetic algorithm with a gradient-based search method can help to overcome some of the limitations specific to the genetic algorithm. The hybridization can help to improve the solution search space with every iteration, thereby reducing the computation time.

### 1.3. Objective

Using RSM and GA collectively for chemical process data analysis can help to overcome the limitations of RSM and reach the global optimization process conditions for the desired product properties. In fact, Shankar et al. [2,5], Tumuluru [20], and Tumuluru and McCulloch [21] have successfully used RSM and GA in combination for optimizing the biomass flow in a single screw extruder, optimization of feed, and fuel properties. For optimization, the ideal algorithm will have the best properties of both stochastic and deterministic optimization. An ideal algorithm would search the entire variable space, while still ensuring local convergence and should scale appropriately with dimensionality. These properties are attainable by combining both stochastic and deterministic algorithms into a single hybrid algorithm.

The overall objective of the present research is to develop a hybrid genetic algorithm by combining a genetic algorithm with a gradient search method to optimize complex processes. The specific objective of the present research is to test the new algorithm on a benchmark and other optimization problems in the areas of food, biofuel and biotechnology research. In the present research, the new algorithm developed was tested on four different functions. The optimization functions were taken from previously published literature: a) Ackley benchmark function and b) functions developed for anthocyanin yield [13], fatty acid methyl ester (FAME) yield [14], and xylanase activity [15].

## 2. Materials and Methods

### 2.1. Hybrid Genetic Algorithm

**Figure 2:**
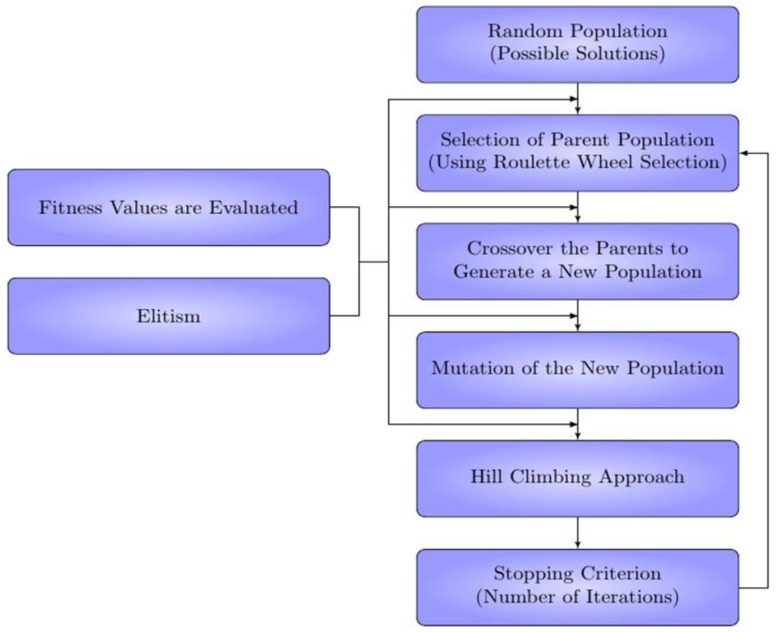
Flow diagram of the hybrid genetic algorithm (HGA) developed at Idaho National Laboratory.

An HGA combines the exploration of a stochastic GA with the exact convergence of a deterministic algorithm [21]. The novelty is how these algorithms are combined. HGA has all of the steps of a regular GA (e.g., selection, mating, mutating, elitism), however, after the mutation routine, each candidate is optimized locally, as the flowchart for the algorithm shows in Figure 2. The combination of these two algorithms leads to an aggressive optimization routine that is capable of exploring the entire variable space and at the same time can converge on the exact local optimum for each candidate. In essence, for a hybrid GA, the placement is governed by natural selection where the best candidate is more likely to determine the placement of new candidates. The main benefit is the ability to extract global optimum values that traditional stochastic algorithms are not capable of detecting. This point is illustrated below with a benchmark and multiple case studies.

### 2.2. Hybrid Genetic Algorithm (HGA) Tool

The HGA uses both the genetic algorithm and steepest ascent hill climbing methods to reach an optimum solution. While hybrid genetic algorithms have gained some attention in recent years [22,23], there is no commercially used software available that can solve the single- and multi-objective optimization problems with various operators, such as tolerance, persistence, applying weights to the objective function, solving objective function by laying constraints, and plotting a surface plot for the objective functions tested. In this research, we have developed a hybrid genetic algorithm optimization tool on the MATLAB platform with an accompanying graphical user interface. This tool was tested to optimize benchmark optimization function (Ackley) and other functions developed in food and bioprocess engineering research. Various algorithm parameters, which can be tested in the HGA optimization software, are given in Section 2.3.

### 2.3. Algorithm Parameters for Single and Multivariable Optimization Problems

*Population:* Population defines the number of candidate solutions to consider for each generation.

*Elitism*: Defines the top percentage of parent solutions to transfer to the child generation.

*Crossover*: Defines the percentage of the child population to generate from breeding from the parent generation. The remaining child population is copied directly from the parent generation. Parents to be copied are selected using the roulette wheel probability method. Parents that result in fitness values closer to the goal (maximum or minimum) are more likely to be copied or used as parents.

*Mutation*: Defines the percentage of the child population to mutate. The alleles of the chosen children are completely randomized. The most elite or fit solution is not a candidate for mutation.

*Iterations*: Is the number of generations to create.

*Lower and Upper Constraints*: The lower and upper constraints apply a bound to the results. Constraints are imposed during the roulette wheel selection subroutine. A given constraint is assigned a weight value, which is used to weight the deviation from the bounds of the results. Using this approach, a user can decide which objective functions are more important to obtain within the given bounds. Note: the weight values for the constraints are not the same weight values for the objective functions.

*Tolerance*: The tolerance determines which solutions are returned as possible answers. If a candidate has a fitness that is within a certain distance from the optimum solution, it is included in the solution set.

*Bounds*: Lower bound and upper bound are vectors that define the limit for the independent variables. In the single objective example these define the limits for x as:

Lower bound < *x* < Upper bound

For the multi-objective optimization example the limits are defined using bounding vectors as:

LB1 ≤ *x_1_* ≤ UB1

LB2 ≤ *x_2_* ≤ UB2

LB3 ≤ *x_3_* ≤ UB3

where the lower bound = (LB1 LB2 LB3) and the upper bound = (UB1 UB2 UB3).

*Goal*: The goal defines whether to maximize or minimize the fitness function(s).

*Weights*: Weights can be assigned to the output of the functions to define importance values for each function in reference to the others.

### 2.4. Case Studies for Testing the Performance of the Hybrid Genetic Algorithm

The HGA was used on the Ackley benchmark function for validation and subsequently on the food processing [13], biofuel [14], and biotechnology [15] case studies. These authors used canonical analysis of response surface methdology, and sequential programming methods to optimize their objective functions. Table 1 indicates the hybrid genetic algorithm parameters used to optimize the benchmark and case studies. Crossover, mutation and elitism rates were optimized for the Ackley benchmark before being applied to the case studies. Parameter rates are based on typical values taken from literature [24].

The input values for the equations developed in foods, biofuels and biotechnology are coded based on the experimental design followed [13,14,15]. The coded values represent the parameter levels for which the experiments were performed. All of the experiments were performed using a full factorial design and optimized using statistical methods such as response surface methodology.

#### 2.4.1. Ackley Benchmark

The benefit of hybridization is highlighted by applying the HGA to a complex benchmark function. For this comparison, the Ackley benchmark function was chosen [25]. The Ackley function is a unique optimization benchmark in the fact that there are many local extrema that are not dramatically better than the surrounding peaks. Deterministic methods get trapped in the local extrema, while the gradual slope of the peaks discourages exploration of the stochastic algorithms. To showcase the benefit of hybridization, that is combining both deterministic and stochastic algorithms, we consider the two-dimensional (2D) Ackley function given in Equation (1) with constants as shown in Table 2. The analytical solution is a null candidate with all zeros. The goal is to minimize this function with input bounds of $-20\leq x_{1}\leq 20$ and $-20\leq x_{2}\leq 20$. Results for the Ackley benchmark are given in Section 3.1.

(1) $$f\left(x_{1},x_{2},\ldots ,x_{n}\right)=-a\exp\left(-b\sqrt{\frac{1}{n}\overset{n}{\underset{i=1}{\sum }}x_{i}^{2}}\right)-\exp\left(\frac{1}{n}\overset{n}{\underset{i=1}{\sum }}\cos\left(c\;x_{i}\right)\right)+a+\exp$$

#### 2.4.2. Foods

Food engineering frequently calls for optimization of numerical models. Consider the anthocyanin yield of purple sweet potatoes [13]. Anthocyanins are beneficial as flavonoids and pigments due to their non-teratogeneses, non-carcinogenicity, non-mutation, and low-ecological impact. Liu et al. [13] developed a surface response model to predict the anthocyanin yield of purple sweet potatoes. The model input parameters are liquid-to-solid ratio (mL/g), ethanol concentration (*w*/*w*, %), ammonium sulphate concentration (*w*/*w*, %), and pH value. The upper and lower limits, along with coded values, are given in Table 3. The goal of this function is to maximize the anthocyanin yield based on the input variables.

(2) $$Y=85.96-1.66x_{1}-1.81x_{2}+1.95x_{3}-8.76x_{4}-0.5x_{1}x_{2}-7.31x_{1}x_{3}-0.33x_{1}x_{4}-0.19x_{2}x_{3}-0.99x_{2}x_{4}-0.93x_{3}x_{4}-2.87x_{1}^{2}+2.25x_{2}^{2}-4.11x_{3}^{2}-8.07x_{4}^{2}$$

*Y* is the anthocyanin yield (%), *x*_1_ is the liquid-to-solid ratio, *x*_2_ is ethanol concentration (%), *x*_3_ is ammonium sulphate concentration (%), and *x*_4_ is pH value. Results for the foods case study are given in Section 3.2.

#### 2.4.3. Biofuel

Biofuel is gaining attention and support due to the volume of global fuel consumption. Eco-friendly alternatives to fossil fuels are being sought to reduce the dependence on finite resources and simultaneously secure national resource independence. Corn-based biodiesels have grown in the last two decades; however, there is a noticeable pressure in the food farming market as subsidized corn crops are going to biodiesel instead of human consumption. Lee et al. [14] developed a model to predict biodiesel yields from the Jatropha curcas plant by considering the reaction time (h), methanol/oil molar ratio, reaction temperature (°C), and amount of CaO-MgO mixed oxide catalyst (wt. %). The upper and lower limits, along with coded values, are given in Table 4. The goal of this function is to maximize the fatty acid methyl ester (FAME) yield based on the input variables.

(3) $$Y=85.17+2.54x_{1}+2.29x_{2}+17.37x_{3}+7.21x_{4}+0.10x_{1}^{2}-0.36x_{2}^{2}-8.49x_{3}^{2}-4.74x_{4}^{2}+0.19x_{1}x_{2}-4.06x_{1}x_{3}-2.56x_{1}x_{4}+0.94x_{2}x_{3}+0.19x_{2}x_{4}-9.31x_{3}x_{4}$$

*Y* is the FAME yield, *x*_1_ is reaction time (h), *x*_2_ is methanol/oil molar ratio, *x*_3_ is reaction temperature (°C), and *x*_4_ is catalyst amount (wt. %). Results for the biofuel case study are given in Section 3.3.

#### 2.4.4. Biotechnology

Enzymes play a crucial role in many biotechnological applications such as breaking down hemicellulose for biofuel production, bleaching wood pulp for paper production, extracting oils and starches and more. The activity of an enzyme in a particular application is of vital importance, as more activity means less usage, which reduces the cost of enzyme use. Vimalashanmugam and Viruthagiri [15] developed a model to predict the production of the xylanase enzyme using a wheat bran substrate. The model input parameters are substrate concentration (g), temperature (°C), incubation time (h), initial moisture content (%), and initial pH value. The upper and lower limits, along with coded values, are given in Table 5. The goal of this function is to maximize the production of the xylanase enzyme based on the input variables.

(4) $$Y=525.67+21.55x_{1}+25.73x_{2}+34.15x_{3}+18.05x_{4}+23.95x_{5}-30.22x_{1}^{2}-29.74x_{2}^{2}-30.53x_{3}^{2}-20.58x_{4}^{2}-29.89x_{5}^{2}-17.02xx_{2}-5.11x_{1}x_{3}+9.75x_{1}x_{4}+5.07x_{1}x_{5}-9.85x_{2}x_{3}+11.33x_{2}x_{4}+1.31x_{2}x_{5}+2.09x_{3}x_{4}+6.77x_{3}x_{5}-0.57x_{4}x_{5}$$

*Y* is xylanase activity, *x*_1_ is substrate concentration (g), *x*_2_ is temperature (°C), *x*_3_ is incubation time (h), *x*_4_ is initial moisture content (%), *x*_5_ is initial pH. Results for the biotechnology case study are given in Section 3.4.

## 3. Results

### 3.1. Ackley Benchmark

**Figure 3:**
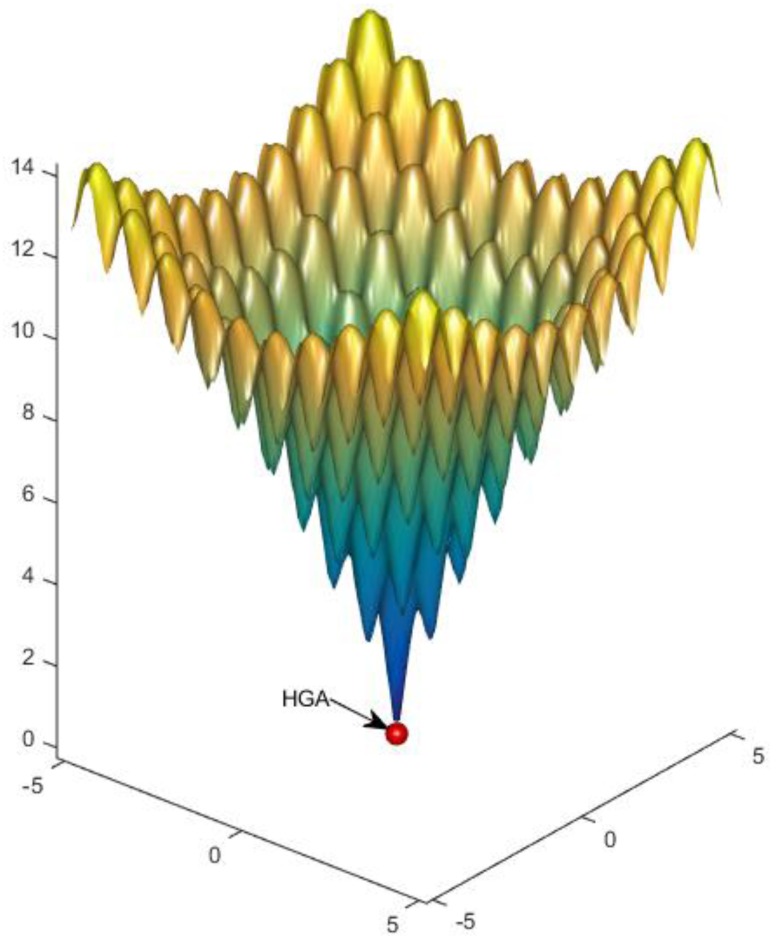
Ackley benchmark function with two independent variables and *n* = 2, a = 20, b = 0.2, c = 2π.

For the Ackley function, the known optimum candidate is all zeros and the corresponding optimum fitness is zero. The optimum value obtained by the hybrid genetic algorithm was 9.0328 × 10^−13^, which indicates that the hybrid genetic algorithm is suitable for multi-dimensional optimization. The graphical representation of the Ackley results is indicated in Figure 3. Based on this result, the remaining case studies were optimized using the hybrid genetic algorithm. Results were compared to the analytical optimum values obtained by the researchers.

**Figure 4:**
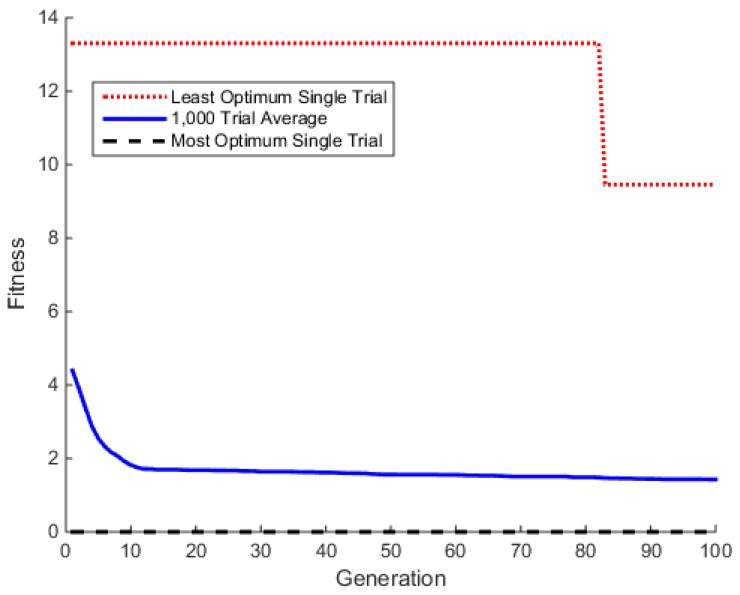
Ackley benchmark profile for 1000 trials with two independent variables and *n* = 2, a = 20, b = 0.2, c = 2π.

Figure 4 shows the profile for the converged solution of the Ackley benchmark using the HGA. To create this profile, 1000 trials, or simulations, were run with the default HGA settings given in Table 6. The average of all of the trials was plotted, as well as the single trial from the 1000 that converged the fastest and slowest.

### 3.2. Food

**Figure 5:**
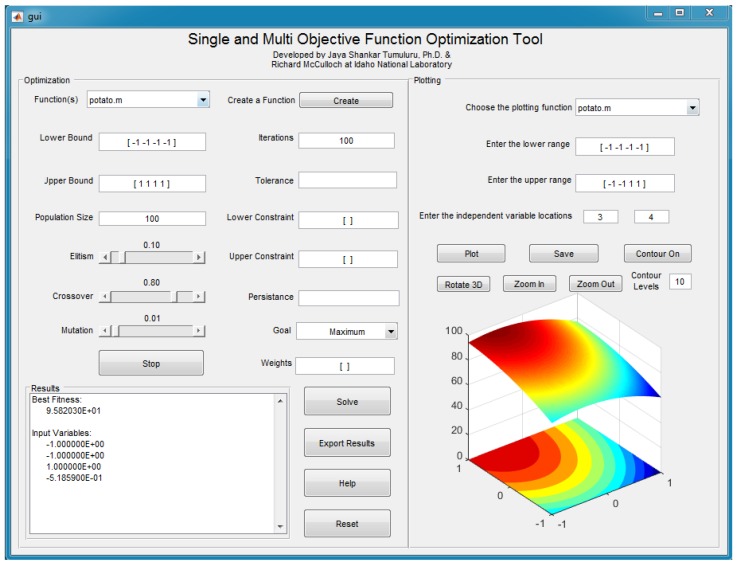
The user front-end of the Multi-Objective Optimization Tool.

The maximum anthocyanin yield reported by Liu, et al. was 90.02% [13]. The HGA converged on an optimum of 95.82%, increasing the yield by more than 5% (see Table 7). Figure 5 is the graphic user interface output of the hybrid genetic algorithm software developed by the authors. The optimum candidate has a 40:1 liquid-to-solid ratio, 23% ethanol concentration, 22% ammonium sulphate concentration, and a pH of 3.2407. By increasing the yield, more product can be derived, bringing profits up and waste down. In Figure 5 the input variables are the coded values between −1 and 1. Also, because the problem is a single objective, the tolerance, constraints, persistence, and weights were not used in solving the optimization problem.

The results indicate that the liquid-to-solid ratio, ethanol concentration, and pH value all have a significant impact on the yield of anthocyanin. Considering process conditions, the hybrid genetic algorithm predicted a lower liquid-to-solid ratio, lower ethanol concentration, and pH needed to maximize the anthocyanin yield. In the bulk production of the anthocyanin yield, the process conditions predicted by hybrid genetic algorithm can reduce the cost while simultaneously maximizing the anthocyanin yield.

### 3.3. Biofuel

Lee, et al. [14] reported that the maximum yield of biodiesel was 93.55%. The HGA converged on an optimum of 98.28%, increasing the yield by more than 4%. The optimum candidate has a reaction time of 2 h, a 40:1 methanol/oil molar ratio, 120 °C reaction temperature, and a catalyst amount of 3.0686 wt. % (see Table 8). Increasing the extraction efficiency lowers the overall cost for biofuel production.

The results show that reaction temperature and catalyst amount play a major role in the biodiesel yield. Naturally, as temperature increases, the cost of processing goes up due to higher energy requirements. Increasing the catalyst amount is also very costly, which presents a trade-off between production yield and cost. Also, reducing the reaction time is important as it can impact the throughput of the reactor. The optimum maximum, predicted by the hybrid genetic algorithm, is approximately a 7% (wt. %) reduction in the catalyst requirement and a 1 h reduction in the reaction time. Lowering both process variables can have an impact on lowering the cost of biodiesel production.

### 3.4. Biotechnology

Vimalashanmugam and Viruthagiri [15] suggested that the maximum xylanase yield under the given conditions was 553.17 IU/gds. The HGA converged on a value of 555.35 IU/gds. The optimum candidate has a substrate concentration of 10.71 g, a temperature of 32.76 °C, incubation time of 133.12 h, initial moisture content of 83.23%, and an initial pH value of 5.25 (see Table 9). Enzymes are expensive to manufacture. Increasing the yield of enzyme production facilities can lower the consumer costs, leading to increased usage and market security for enzymatic applications.

The results obtained by the HGA are marginally higher to those reported by Vimalashanmugam and Viruthagiri [15]. The benefit of using a rudimentary deterministic algorithm, such as steepest descent, is the ability to converge on a minimum using adaptive steps. This allows the solution to be refined to a tolerance specified by the user. The results show that the HGA was used with tighter tolerances, which led to small deviations to improve the solution.

## 4. Discussion

Optimization is crucial in analyzing experimental results and models. Stochastic optimization performs well where deterministic optimization fails. Problems with high dimensionality or very large variable spaces become impossible for deterministic methods to solve in a reasonable amount of time. Stochastic algorithms can search the variable space and dynamically bias the candidate generation toward the optimum solution. Stochastic algorithms are also crucial in situations where deterministic methods cannot be applied, for example when a machine part will fail, optimum critical path problems, decay fission products, etc. GAs utilize stochastic operations such as crossover and mutation on a population to make a change of generation. Crossover combines substructures of parents to produce new individuals. Crossover is the core of the genetic algorithm and is what sets it apart from other stochastic methods, such as simulated annealing [25]. Mutation is another operation that helps the algorithm to prevent local convergence and search the global variable space. A simple genetic algorithm with operators such as crossover, mutation, and elitism yields goods result in practical optimization problems compared to a deterministic algorithm [2,5]. Not much work has been done on understanding the effect of the genetic algorithm operators such as crossover, mutation, and elitism on the function convergence.

Stochastic algorithms have some inherent drawbacks when compared to deterministic methods. First and foremost is the fact that the exact optimum is never actually achieved [26]. The optimum candidate can be very close to the actual optimum, however, there is no way of knowing for certain that the optimum candidate is at the global maximum or minimum. Another potential problem is the rate of convergence. For some algorithms, such as simulated annealing, tuning parameters allow for rapid or reduced convergence [27]. If the algorithm is forced to converge too quickly, the chances of attaining the optimum become very low as the system converges on a chaotic state. One of the biggest disadvantages of using a genetic algorithm is that the GA cannot assure constant optimization response times. This unfortunate genetic algorithm property limits the use of genetic algorithms in real-time applications. Certain optimization problems (called variant problems) cannot be solved by means of genetic algorithms. This is mainly due to poorly known fitness functions that generate bad chromosome blocks in spite of the fact that only good chromosome blocks cross over. On the other hand, the hybridization of the genetic algorithm with a deterministic algorithm helps to overcome these problems and to achieve the globally optimum solution applicable to any industry that requires optimization, especially for problems with high dimensionality and large domain spaces that are not readily solved by traditional deterministic or stochastic algorithms. This study has proved the hypothesis that hybridization of the genetic algorithm with a deterministic algorithm improves the optimum solution obtained by statistical methods alone. This hybrid genetic algorithm can be successfully applied to various processes and achieve better optimized results compared to traditional methods. Our current research work is on generating a Pareto front using the hybrid genetic algorithm. By introducing an objective function, a series of viable solutions can be determined that can optimize the yield of the process. The Pareto front generated for an objective function can help the process managers make decisions on what optimal conditions they are interested in running in order to maximize the yield or minimize the cost.

## 5. Conclusions

The present research was on understanding the effect of hybridization of a genetic algorithm with a gradient-based method on function optimization. The hybrid genetic algorithm developed was tested on optimizing the Ackley benchmark optimization function, the anthocyanin yield, fatty acid methyl ester (FAME) yield, and xylanase activity functions published in the literature. The minimum value obtained using the hybrid genetic algorithm for the Ackley function was 9.0328 × 10^−13^. In food processing, the maximum anthocyanin yield obtained was 95.82% compared to the literature value of 90.02%. The maximum anthocyanin yield was achieved at a lower liquid-to-solid ratio and ethanol concentrations. In biodiesel production, the hybrid genetic algorithm predicted a maximum of 98.28% compared to the source value of 93.55% obtained using a canonical analysis of the response surface method. The hybrid genetic algorithm optimum indicated that these higher yields are achievable at reduced catalyst and reaction time. In enzyme production, the HGA predicted a maximum yield of 555.35 IU/gds, where the literature value was 553.17 IU/gds obtained using an RSM analysis. The results show that the hybrid genetic algorithm predicted better optimized process conditions and product yields compared to regular statistical methods.

## Figures and Tables

**Table 1:** Parameters used for the HGA based on typical values found in literature.

Population Size	Generations	Crossover	Mutation	Elitism
100	100	0.80	0.01	0.10

**Table 2:** Ackley benchmark constants.

Dimensions	a	b	c	Range
2	20	0.2	2π	−20 < x < 20

**Table 3:** HGA variable coding for the food case study.

Parameter	$x_{1}$	$x_{2}$	$x_{3}$	$x_{4}$
Upper Limit (coded value)	60 (1)	27 (1)	22 (1)	4 (1)
Lower Limit (coded value)	40 (−1)	23 (−1)	20 (−1)	3 (−1)

**Table 4:** HGA variable coding for the biofuel case study.

Parameter	$x_{1}$	$x_{2}$	$x_{3}$	$x_{4}$
Upper Limit (coded value)	4 (1)	40 (1)	120 (1)	4 (1)
Lower Limit (coded value)	2 (−1)	10 (−1)	80 (−1)	2 (−1)

**Table 5:** HGA variable coding for the biotechnology case study.

Parameter	$x_{1}$	$x_{2}$	$x_{3}$	$x_{4}$	$x_{5}$
Upper Limit (coded value)	5.2 (2.378)	27.2 (2.378)	62.9 (2.378)	68.1 (2.378)	3.8 (2.378)
Lower Limit (coded value)	14.8 (−2.378)	36.8 (−2.378)	177.1 (−2.378)	91.9 (−2.378)	6.2 (−2.378)

**Table 6:** Results for the Ackley benchmark case study.

Case Study	Optimum Candidate		Optimum Minimum	
HGA	*x*_1_	2.4869 × 10^−13^	F	9.0328 × 10^−13^
	*x*_2_	−1.9895 × 10^−13^		
Analytical	*x*_1_	0	F	0
	*x*_2_	0		

**Table 7:** Results for the case study on the anthocyanin yield.

Case Study	Optimum Process Conditions		Optimum Maximum	
HGA	Liquid/Solid Ratio	40	Anthocyanin yield	95.82
	Ethanol concentration	23		
	Ammonium sulphate	22		
	pH value	3.24		
Liu, et al. [13]	Liquid/Solid Ratio	45	Anthocyanin yield	90.02
	Ethanol concentration	25		
	Ammonium sulphate	22		
	pH value	3.30		

**Table 8:** Results for the case study on the biodiesel yield.

Case Study	Optimum Process Conditions		Optimum Maximum (%)	
This work (hybrid genetic algorithm)	Reaction time (h)	2.00	Biodiesel Yield	98.28
	Methanol/Oil Molar Ratio	40.00		
	Reaction temperature (°C)	120.00		
	Catalyst amount (wt. %)	3.07		
Lee, et al. [14]	Reaction time (h)	3.44	Biodiesel Yield	93.55
	Methanol/Oil Molar Ratio	38.67		
	Reaction temperature (°C)	115.87		
	Catalyst amount (wt. %)	3.70		

**Table 9:** Results for the case study on the xylanase yield.

Case Study	Optimum Process Variables		Optimum Maximum (IU/gds)	
This work (hybrid genetic algorithm)	Substrate concentration (g)	10.71	Enzyme Yield	555.35
	Temperature (°C)	32.76		
	Incubation time (h)	133.12		
	Initial moisture (W)	83.23		
	Initial pH	5.25		
Vimalashanmugam and Viruthagiri [15]	Substrate concentration (g)	10.70	Enzyme Yield	553.17
	Temperature (°C)	32.70		
	Incubation time (h)	133.00		
	Initial moisture (W)	83.20		
	Initial pH	5.30		

## Abbreviations

The following abbreviations are used in this manuscript:

GA	Genetic Algorithm
HGA	Hybrid Genetic Algorithm
RSM	Response Surface Methodology
FAME	Fatty Acid Methyl Ester
